# No More Sneaking Around: The Changing Terminology of Orangutan Male Reproductive Strategies

**DOI:** 10.1002/ajpa.70252

**Published:** 2026-07-16

**Authors:** Alexandra E. Kralick

**Affiliations:** ^1^ Department of Gender and Women's Studies and Department of Anthropology University of Wisconsin‐Madison Madison Wisconsin USA

**Keywords:** bimaturism, feminist science studies, orangutans, alternative reproductive tactics, terminology

## Abstract

**Objectives:**

Orangutans exhibit male bimaturism, characterized by pronounced variation among adult males. Early research framed this variation as age‐based morphs with two alternative reproductive tactics (ARTs), contrasting “sneak and rape” subadults with “consort and combat” adults. Subsequent research demonstrated that males previously labeled as subadults can remain unflanged well into adulthood, and both types of males mate with and without force, leading to a revised ART framework: flanged males “sit, call, and wait” while unflanged males “go, search, and find.” This study examines how these frameworks have been taken up and represented over time in the scholarly literature.

**Materials and Methods:**

A bibliometric analysis of 810 publications which use orangutan and morph‐ and strategy‐based keywords identified 124 relevant publications which were coded by morph and strategy classification and analyzed for temporal trends.

**Results:**

The “subadult” and “sneak and rape” terminology is no longer dominant in peer‐reviewed primatology; yet, these imprecise frameworks persist, particularly outside of primatology in the secondary literature regarding the evolution of human male aggression.

**Discussion:**

The persistence of the “sneak and rape” framework does not reflect empirical evidence, as primatology now distinguishes male morphs without defining them by sexual aggression. Nor can it be explained by disciplinary lag alone, as some studies update the term from subadult to unflanged male while retaining outdated behavioral framing. Instead, selective uptake aligns with broader narratives in human evolution that naturalize male aggression and female vulnerability, underscoring the need to clarify primatological frameworks that inform interpretations of human evolution.

## 1 Objectives

Evolutionary models of human sexuality often draw on primatology and animal behavior as an evidence base (e.g., Gray 2013; Martin 2007; Miller 1931; Silk 1993; Smuts 1992, 1995). Feminist scholarship has critiqued such models when they prioritize male competition and dominance while marginalizing or oversimplifying female roles, often portraying them as passive (Fedigan 1986; Gowaty 1997; Hrdy 1999; Tang‐Martínez 2016). These critiques highlight female behavioral variability and active reproductive strategies, challenging traditional paradigms and advocating for a more comprehensive understanding of sexual evolution (Haraway 1989; Hrdy 1999; Sperling 1991). Consequently, a wide range of female variation in non‐human primates, including dominance and active reproductive roles, is now recognized (Drea 2005; Drea and Davies 2022; Lewis 2018, 2019; Lewis et al. 2023). However, a comparable range of male variation in non‐human primates, including non‐dominant roles, remains underexplored. Here, I highlight a form of male variation in one genus of non‐human primate, the orangutan.

Male orangutans display such a remarkable range of variation that they are now recognized as having two distinct types, or morphs, flanged and unflanged, characterized as exhibiting “Alternative Reproductive Tactics” (ARTs) (Atmoko et al. 2009; Kunz et al. 2023; Scott et al. 2024). This variation was first detailed by field researchers five decades ago (Galdikas 1979; MacKinnon 1974; Rijksen 1978), and since then, the characterization of male orangutan variation and associated reproductive tactics has changed substantially. In response to new data, one of the male morphs was changed in characterization from “subadults” who “sneak” and force copulations to adult unflanged males who “go, search, and find” mates (Utami et al. 2002).

The goals of this paper are to (1) provide clarity regarding the shifting framings over time of nondominant orangutan males, in particular the shift away from 'subadult' “sneak and rape” terminology toward unflanged and “go, search, and find” terminology, (2) examine when and where this shift has or has not been taken up in the academic literature, (3) bring attention to the stakes of how male reproductive strategies are framed for broader discussions in the evolution of human behavior, and (4) highlight existing primatological terminology which offers an opportunity to move beyond sexual aggression as the defining feature of within‐male difference and instead move towards terms that reflect the full breadth of within‐male variation observed in wild orangutans.

### 1.1 Orangutan Bimaturism

**FIGURE 1:**
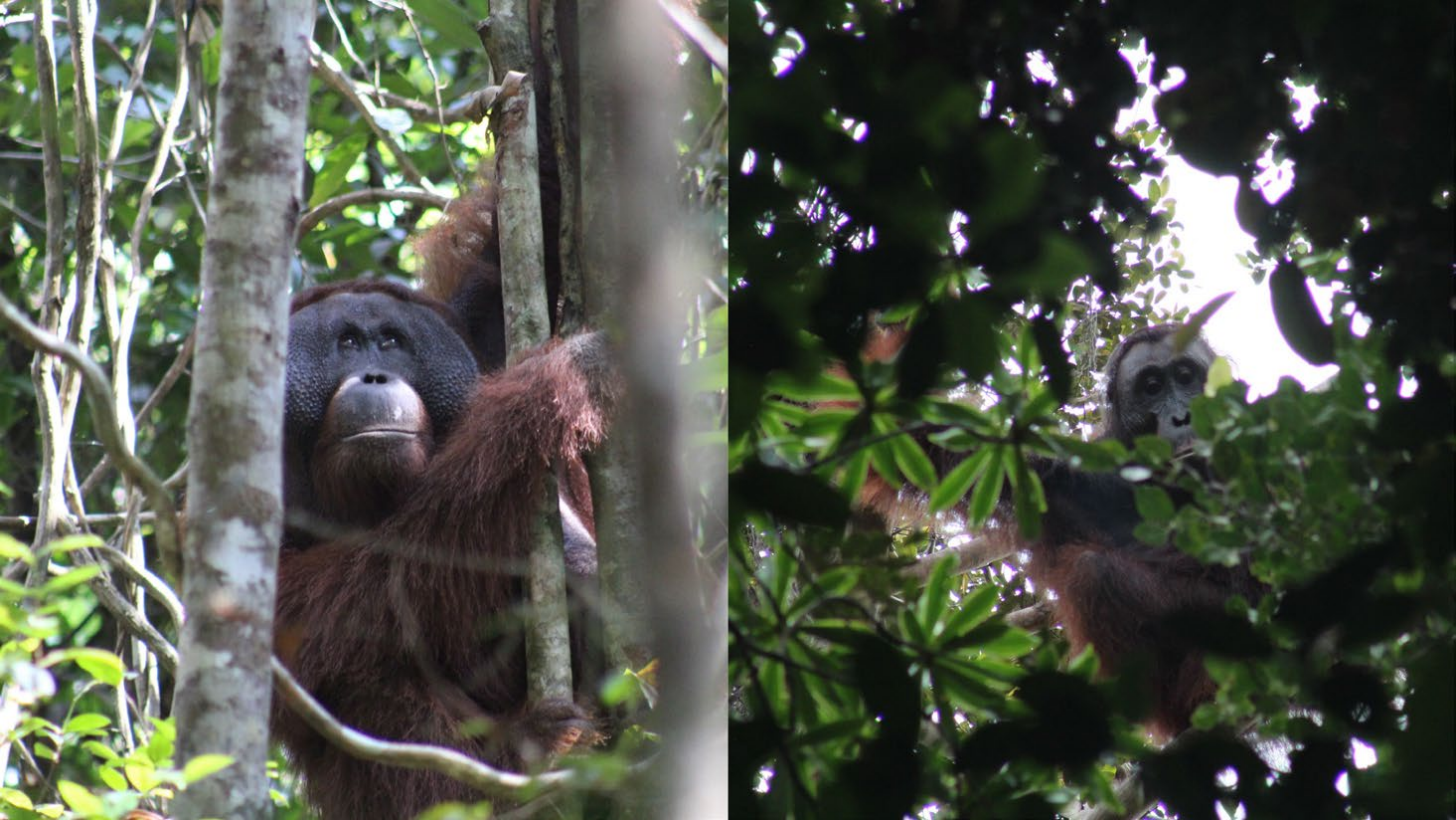
Flanged male on left, named Momo. Unflanged male on right, named Suan. Tuanan Orangutan Research Station, Central Kalimantan, Borneo. *Source*: Photos by author.

Orangutan males exhibit a unique form of variation, whereby some males fully develop secondary sexual characteristics, such as cheek pads or flanges and laryngeal throat sacs or throat pouches for making long calls, while others remain without these features while reproductively active (Figure 1). The former are known as flanged males for their characteristic large cheek pads called flanges (Atmoko et al. 2009; Kuze et al. 2005; Pradhan et al. 2012) and are the adult males most people are familiar with when they think of adult male orangutans. In contrast, the latter are lesser‐known, as naturally occurring delays in the development of secondary sex characteristics beyond age 20 have only been documented in wild populations.^1^ Early Western scientists^2^ were puzzled by the presence of these lesser‐known males, postulating that because they were smaller and lacked a full suite of secondary sex characteristics, they comprised a different species of orangutan associated with yet unseen smaller females (Wallace 1856; Lyon 1908), an idea which may go back to Petrus Camper in the 1700s (Meijer 1991, 2014). It was not until the 1970s, with the establishment of long‐term field research, that researchers were able to confirm that both morphs belong to the same species.

### 1.2 Early Framings‐ Subadult Male “Sneak and Rape” and Adult Male “Consort and Combat”

Early field researchers of orangutans described variation in male secondary sex characteristics as different developmental stages each individual went through; first a smaller (see Box 1) ‘subadult’ phase where males are reproductively active but lack secondary sex characteristics, then a second phase after puberty, the ‘adult’ phase, in which males have developed their secondary sex characteristics (Galdikas 1979, 1988; MacKinnon 1974). MacKinnon (1974) was the first to describe behavior in the ‘subadult’ phase. He observed ‘subadult’ males to be the only ones who mated, witnessing no adult males mate. Based on these findings, MacKinnon suggested that orangutan reproductive behaviors are ‘out of phase,’ meaning ‘subadult’ males are primarily responsible for reproduction whereas adult males function as territorial guardians (MacKinnon 1974). Subsequently, Rijksen (1978) observed the opposite pattern, whereby ‘subadult’ males mated less frequently than adult males. Rijksen proposed that ‘subadults’ occasionally engage in “rape” while adult males do not. Galdikas built off the terminology of MacKinnon and Rijksen to form the basis of what is today known as the orangutan alternative “reproductive strategies” framework (Galdikas 1985a).

Galdikas proposed that male orangutans exhibited two different reproductive strategies: the ‘subadult’ male strategy of “sneak/rape” and the adult male strategy of “consort/combat” (Galdikas 1985a). Having observed aggression and combat between adult males, she noted that considerable energy had to be expended to mate. Thus, Galdikas postulated that adult males are primarily interested in attracting and guarding receptive females, as adult females are relatively selective in their choice of consort partner, calling this strategy “consort/combat.” This was in contrast to ‘subadult’ males, whom she observed 19 times forcibly copulating with non‐receptive females, as opposed to once by an adult, thus giving rise to the framing “sneak/rape” (Galdikas 1985a).^3^ The papers that followed after Rijksen and Galdikas built on the idea without reiterating the term “rape”, instead describing the phenomenon as “forced copulations” (Fox 1998; Schürmann and van Hooff 1986). Nadler acknowledges the shift as a response to “admonitions against using anthropomorphic terminology to describe behavior in animals” (Nadler 1988), citing literature around the time that described using the emotionally charged word rape for nonhuman animals as anthropocentric, implying motivation, and causing confusion and misunderstanding (e.g., Estep and Bruce 1981).^4^ Subsequently, papers examined orangutan male reproductive strategies in the context of either female‐choice or male–male competition. Schürmann and van Hooff (1986) and van Hooff and van Schaik (1995) argued that the evolution of two distinct sexually mature male orangutan morphs with different strategies depends on female choice for flanged males. In contrast to the female choice model, Rodman and Mitani (1987) suggested a model of male–male competition, such that a dominant flanged male could not monopolize access to all females (given the solitary nature of orangutans, wide spatial dispersion of females, dense forest habitat, and large home ranges) meaning this opened up a mating opportunity for ‘subadult’ males (van Hooff and van Schaik 1995; van Schaik and van Hooff 1992). It was not until the turn of the century that the framing was flipped by three major pieces of evidence about these so‐called ‘subadult’ males: (1) these males may live well beyond the age of puberty, (2) they sired the majority of offspring in the first paternity study, and (3) they do not exclusively force copulations nor are they the only ones to do so.

### 1.3 Later Framings‐ Unflanged Male “Go, Search, and Find” and Flanged Male “Sit, Call, and Wait”

The first major piece of evidence that shifted the framing around was the reporting that ‘subadult’ males, sexually mature but lacking secondary sexual characteristics, could be found living well beyond the typical onset of puberty at 14 or 15 years (Utami et al. 2002), confirming initial postulations by Rodman and Mitani (1987). Not long after, males were documented without the full suite of secondary sex characteristics at ages well into their twenties, thirties, or even beyond (Knott and Kahlenberg 2007; Utami Atmoko and van Hooff 2004; Wich et al. 2004). Since an elderly male could hardly be called a ‘subadult’, this discovery prompted the realization that the term ‘subadult’ was misleading and that these males should be regarded as adults as well (Utami Atmoko and van Hooff 2004). Instead of being called ‘subadult,’ these males began to be referred to as unflanged males. This term was already in circulation, with van Schaik using it interchangeably with 'subadult' just a few years earlier, noting “subadult (unflanged) males associated with females whose infants were large and about to be weaned, and often mated with them, if necessary by force” (van Schaik 1999). Later, van Schaik and others acknowledged this shift explicitly, stating that “sexually mature males come in two classes: unflanged (formerly usually called subadult) and flanged (formerly known as adult)” (van Schaik et al. 2009, 259). Thus, the discovery of sexually mature males in older ages lacking secondary sexual characteristics led to the reclassification of this group from ‘subadults’ to ‘unflanged' males, reflecting a more accurate representation of the full range of male variation. Today, sexually active adult male orangutans are recognized in two morphs or types: flanged males, who have secondary sex characteristics, and unflanged males, who lack secondary sex characteristics.

BOX 1 'Subadults' or unflanged males have been described to be “female‐sized” (Galdikas 1985a; Fox 2002; Delgado and van Schaik 2000; Rayadin and Saitoh 2009; Banes et al. 2015; Manduell et al. 2011); however, it is now known that unflanged male body sizes exist across a broad range of sizes and that these males are not just “female sized.” As demonstrated in Kralick et al. (2023), the largest dataset of wild unflanged male body sizes published to date, younger adult unflanged males overlap with the female range of body mass, whereas older adult unflanged males are larger than the typical “female size” in body masses and have limb lengths that can overlap with the values of flanged males. This is particularly the case for the unflanged males with older skeletal age determined using third molar dental development via x‐ray assessment (root open/closed) and relative level of dental wear (Kralick et al. 2023). These findings are consistent with measurements of limb length from a wild photogrammetry study, which found that one unflanged male had a limb length that overlapped with the values of the flanged males (Brown et al. 2022) and with an estimated lean body mass study, which found that the urinary creatinine levels of 13 unflanged males fell mostly in the range of adult females or between adult females and flanged males, with at least one unflanged male solidly in the flanged male range (see figure 1 of Harwell et al. 2026). That said, it is worth noting that the male and female ranges were not dichotomous; while they significantly differed, there were adult females with urinary creatinine levels in the characteristic flanged male range and adult flanged males with levels overlapping with those of the adult females (Harwell et al. 2026). Overall, it is possible that the first orangutan males described as 'subadult' were in fact “female sized” because they were younger unflanged males, whereas older unflanged males can exhibit body size measures above the typical female range.

The second major finding is that male paternity is heterogeneous, with unflanged males having a variable degree of reproductive success. The first paternity study found that three unflanged males fathered 6 out of 10 offspring, while four offspring were sired by three flanged males (Utami et al. 2002). All subsequent paternity studies have found unflanged males to have lower paternity rates than flanged males (Banes et al. 2015; Goossens et al. 2006; Tajima et al. 2018; van Noordwijk et al. 2023), including the first study of a population with no formerly captive individuals (Scott et al. 2024), providing evidence that unflanged males exhibit a form of conditionally dependent alternative reproductive strategy, whereby the less successful tactic is thought to “make the best of a bad job” (Dawkins 1980; Gross 1996). Still, all these studies found both flanged and unflanged males sire offspring. Further, there are times and situations in which unflanged males do have a measure of reproductive success. For example, unflanged males may have higher reproductive success in times of hierarchy instability (Banes et al. 2015).

The third major body of evidence that caused “sneak and rape” to be dropped was that unflanged males do not exclusively force copulations, nor are they the only ones to do so. Flanged males are also reported to force copulations, and unflanged males engage in cooperative matings (Knott et al. 2010). In fact, a 2010 study by Knott and others found that flanged males, or “prime males,” were more likely to use “aggression or physical restraint in their mating interactions,” suggesting “intimidation” is also used as a flanged strategy. The finding that the flanged males also force copulations is not a recent one; the first Western scientist to observe an orangutan copulation described an adult male (what we now know to be a flanged male) copulating with a female in a way that was “violent and brief” (Horr 1975). MacKinnon (1979) never witnessed sexual behavior involving a flanged male but did observe both forced and unforced matings by unflanged males. Referencing MacKinnon's study, Drea and Wallen (2003) note that “sub‐adult males often engage in consortships with females, implying that the dichotomy between unflanged “rapists” and flanged consorts may not be as clear as previously suggested.” Mitani also found this pattern in his 1985 study, reporting 144 forced and 7 unforced matings by small males and 13 forced and 15 unforced matings by large males (and a significantly longer median length of association by small males) (Mitani 1985a, 2021). Fox, who observed over 200 orangutan matings, wrote, “the above depiction of male mating aggression [sneak/rape], while perhaps correct, is nonetheless an incomplete account of variation in orangutan sexual behavior” (Fox 1998, 58). As Scott puts it, “Based on early field observations, it was suggested that forced copulations were an alternative mating strategy employed by unflanged males. However, this does not explain the use of force by flanged males that has since been observed in subsequent studies” (Scott 2019). A study of the occurrence of female resisted and voluntary copulations across two sites, Suaq and Tuanan, found that “all in all, these observations support the idea that coercion is a variable and highly context dependent mating tactic, which may be linked to male competition intensity, rather than a morph‐specific strategy or an individual male trait,” concluding that “coercion is neither an ‘unflanged‐default’ nor an individual male trait” (Kunz et al. 2021). A recent study found that “although females were more likely to resist mating attempts by unflanged than by flanged males, sexual coercion is not the default strategy of unflanged males. Unflanged males did not force all their copulations, nor did flanged males refrain from forcing copulations in all contexts” (Kunz et al. 2023). Another study found “male type (flanged or unflanged) was not a significant predictor of the degree of proceptivity displayed by a female” (O'Connell et al. 2020). Forcing copulations is so common across both types of male orangutans that Knott argued that “rather than only being a strategy of unflanged males, forced copulations in orangutans should be seen as simply a male strategy—one that can be used by either flanged or unflanged males to overcome female resistance” (Knott et al. 2010) and that the occurrence of forced copulations is less a male strategy to force and more likely to be a contingent response to female resistance as a result of female choice (Banes et al. 2015; Knott et al. 2010). Regardless, given abundant evidence that forced copulations are highly variable and that both types of males may forcibly copulate, it is clear there was a need to shift away from framing unflanged males as solely employing a strategy of forced copulation.

Thus, given this body of evidence, Utami et al. proposed a new phrase, “going, searching, and finding” (Utami et al. 2002) supported by longstanding evidence that unflanged males travel further per day than flanged males (Mitani 1989; Utami 2000). The idea is not new and goes back to Mitani (Campbell 1992), who wrote in 1985 that orangutan males may either defend a range against other males or range widely, attempting to mate with a greater number of females (Mitani 1985). Confusingly, the term has not exclusively been used to reference reproductive strategies; Morrogh‐Bernard et al. (2009) argued that there are two dietary foraging strategies in orangutans, “sit and wait” and “search and find.” However, the authors did find a general trend (although not statistically significant) of the longest rests by flanged males and the longest travels by unflanged males; this finding supports the idea that unflanged males range more widely (Morrogh‐Bernard et al. 2009). Thus, it is said that “unflanged males range more widely looking for females, hoping for the possible chance that a female agrees to a consortship (‘go‐and‐find’)” (Utami Atmoko and van Hooff 2004, 199). Recent publications continue to reiterate the framing that unflanged males employ a reproductive tactic of “go, search, and find” (Kunz et al. 2023; Scott et al. 2024).

As the framing of unflanged males shifted, so too did that of flanged males. Previously, “consort and combat” or “consort and copulate” was based on the range‐guard and female choice hypotheses. The range guard, as it was postulated by MacKinnon (1974), suggested that only subadult males are reproductive and that adult males are post reproductive and defend a range including subadult males for an inclusive fitness benefit. However, many subsequent studies have shown flanged males to be sexually active (Schürmann 1981; Mitani 1985a; Schürmann and van Hooff 1986). Further, while flanged males do fight and severely injure one another, flanged males are not necessarily territorial (Setia and van Schaik 2007; Pradhan et al. 2012), instead, their ranges overlap (Knott 1998; Singleton and van Schaik 2001; Knott and Kahlenberg 2011; Alavi 2018). Rather than range‐guarding, strong evidence exists for the role of long calls as a hallmark of the flanged tactic. Females will go toward calling flanged males (Setia and van Schaik 2007; Utami and Setia 1995), and there is evidence of flanged males long calling when not in association with females (Delgado et al. 2009). As Utami‐Atmoko and van Hooff (2004, 199) write, “there are flanged males who rely on a female preference and advertise their presence with long‐calls, and wait for females to join them (“long‐call‐and‐wait”)”, also known as “sitting, calling, and waiting” (Atmoko et al. 2009; Utami et al. 2002), with many variants including long‐call‐and‐wait, calling and waiting, call‐and‐wait, sit‐call‐and‐wait, etc. Thus, the idea around framing the strategy of flanged males as “calling and waiting” is that they are making long calls and waiting for females to come to them when they are interested in mating, relying on a variant of female choice.

However, the waiting part of “calling and waiting” has since shown mixed evidence. While there is consistent evidence that long calls support a mate attraction function, this likely involves not just drawing receptive females but coordinating ranging of non‐receptive females (Delgado et al. 2009). Not all sites and playback experiments find support for females approaching male long calling (Delgado 2003; Mitani 1985b) suggesting that long calls have more functions than only attracting females. These involve an intrasexual spacing function among males maintaining a dispersed social network (Delgado et al. 2009). Thus, the idea that females seek protection from sexual coercion via attraction to long calls as a beacon is less widely supported (Delgado et al. 2009, sensu Smuts and Smuts 1993, see Setia and van Schaik 2007). Further, flanged males range widely, as field studies frequently observe new individuals entering the study area, including older flanged males (Spillmann et al. 2017). Flanged males may also search and find, with one study noting “flanged males do not necessarily ‘call and wait’ and instead can ‘search and find’ fertile females” (Hayward 2018). Thus, differentiating flanged males by calling alone may be more accurate than “sit, call, and wait.” Since both flanged and unflanged males show a range of reproductive tactics and behaviors, presence or absence of long calls is a differentiator that has no overlap between males with flanges and throat sacs and those without.

Over the decades, many terms and conceptual framings have been proposed to conceptualize orangutan male bimaturism (Table 1). The main aim of this paper is to improve conceptual clarity and reduce ambiguity and confusion by bringing these terminological transitions to the fore, particularly for research in biological anthropology that references orangutan bimaturism. There is a wide body of literature that draws upon primatological data as evidence to make claims, particularly literature in human evolution, anthropology, evolutionary biology, and evolutionary psychology. Despite the clear recognition within primatology that the framework of “subadult” and “sneak and rape” is imprecise, not empirically supported, failing to capture the breadth of variation within males, and loaded with an anthropocentric term, it has remained unclear whether these frameworks have actually been displaced in the broader literature beyond primatology. This study tested whether, where, and why non‐empirically supported terms continue to appear outside of primatology.

**TABLE 1:** Visual representation of the shifts in definitions of orangutan male morphs over time.

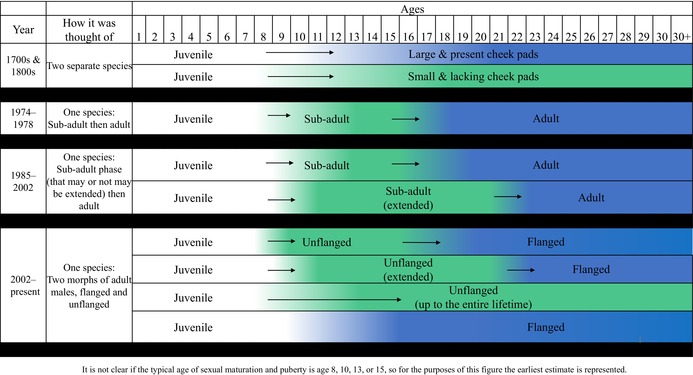

## 2 Materials and Methods

A bibliometric analysis was employed to evaluate shifting conceptualizations of orangutan male bimaturism in the literature over time. Publish or Perish (Harzing 2007) was used to pull all Google Scholar referenced publications which included the word “orangutan” in combination with keywords of either “subadult” or “unflanged” and either “sneak” and “rape” or “force” and “copulations” or “search” and “find.” From within the 810 publications identified (journal articles, books, book chapters, book reviews, bachelor's and master's theses, and dissertations), a total of 124 publications were deemed relevant (Appendix S1). Those relevant publications fell between the years of 1974 and 2025; 39 of them were peer‐reviewed journal articles. Journal title, book title, or dissertation completion department were used to assess disciplinary field.

**TABLE 2:** Flow chart visually representing literature review methodology employed for categorizing papers.

Step	Question	If Yes	If No
1	Does it describe orangutan bimaturism, two morphs of males, subadult vs. adult males, arrested adolescents and adults, arrested males, a different behavior of adolescent vs. adult males, or flanged vs. unflanged males?	Move to step 2	Exclude/irrelevant
2	Does it describe the difference between the two morphs of males in terms of behavior/evolutionary explanation/adaptation/strategy?	Move to step 3	Exclude/irrelevant
3	Does it use the terms “sneak” or “rape,” to describe what differentiates the behavior of subadult, smaller, arrested, adolescent, or unflanged males (when compared to adult, larger, or flanged males, especially when compared to consort/combat behaviors)?	*Code SR* If it also describes subadult/unflanged strategy as that of sexual harassment, bullying, or coercion‐ *Code SH* Move to step 4	Move to step 4
4	Does it unequivocally describe uncooperative or forced copulation events (e.g., resistance, injury, rates of forced vs. cooperative copulation) as what differentiates the behavior of subadult, adolescent, arrested, smaller, or unflanged males from adult, larger, or flanged males?	*Code FC* If it instead states that subadult/smaller/arrested/adolescent/unflanged males are more likely to use force or forced copulation occurs more often but not exclusively‐ *Code FC1* to indicate nuance Move to step 5	Move to step 5
5	Does it describe the behavioral or evolutionary strategy of subadult/smaller/arrested/adolescent/unflanged males as ranging more widely (“search and find”) to get mating opportunities when compared to flanged/large males who “sit, call, and wait” for females to come to them by long calling?	*Code GSF* Does not need to use exact terms of “search and find” or “go search and find” but does need to describe ranging widely versus traveling less as a/the key difference Move to step 6	Move to step 6
6	Does it give a different behavioral and/or evolutionary explanation for bimaturism or the existence of unflanged/subadult males (e.g., differences in sociality and gregariousness, inter‐male aggression without referencing mating, diet)?	*Code O* for other	End

For each publication deemed relevant, pertinent quotes were identified and used to code the manuscript. The publications were coded according to how the males were named and which framings were employed. Options for “name for the males” were subadult (S), unflanged (U), or other (O). Options for “reproductive strategy framing” were “Sneak and Rape” (SR), “Go Search and Find” (GSF), Sexual Harassment (SH), Force Copulations (FC), males who force copulate at higher rates, although not always and/or they are not the only males who force copulate (FC1), as well as one unusual category that mixed the various other terms together of “Sneak and Rape” for unflanged males but “Call and Wait” for flanged males, evoking the other half of “Go Search and Find” (CWSR). The coding was done following a flowchart in (Table 2) and a six‐part code system (Table S1) as outlined in Wutich et al. (2024). Sample sizes are distributed by each code type (Table S2). The same publication could have multiple “reproductive strategy framing” codes but only one “name for the males” code. Twentythree publications were classified as naming these males as subadult while employing the framing of “sneak and rape.” Thirty‐eight publications were classified as naming the males as unflanged while employing the framing of “go search and find”.

Visualization of data and statistical analyses was conducted in R (R Core Team 2023). Proportional usage was calculated as the mean presence of each term within year‐by‐field groupings. Trends in the number of publications per year as well as proportions of publications by year and field were visualized with regression lines and smoothed trends estimated using locally weighted regression (LOESS) to assess change over time. In addition to visual and statistical analysis, quotes were qualitatively analyzed to examine the argument made by the publication and the reasoning for why orangutan male bimaturism was discussed or referenced.

## 3 Results

Results indicate that the framework for male bimaturism has changed substantially over time. The relative balance of different taxonomies has shifted, with prior taxonomies of “subadult” and “sneak and rape” no longer being the prevailing ones within primatology. Specifically, the only publication in the peer‐reviewed primatological literature after 2002 that references “sneak and rape” is a study on a monkey species, not orangutans. However, the imprecise categories of “subadult” and “sneak and rape” persist past 2002 and are found outside of the field of primatology in literature referencing them to make claims about the evolution of human behavior, particularly sexual violence and aggression in men.

*Result 1*: *The taxonomies of “subadult” and* “sneak and rape” *are no longer dominant in the primatological peer‐reviewed literature. However, these terms persist in the broader academic literature, especially outside of primatology*.

**FIGURE 2:**
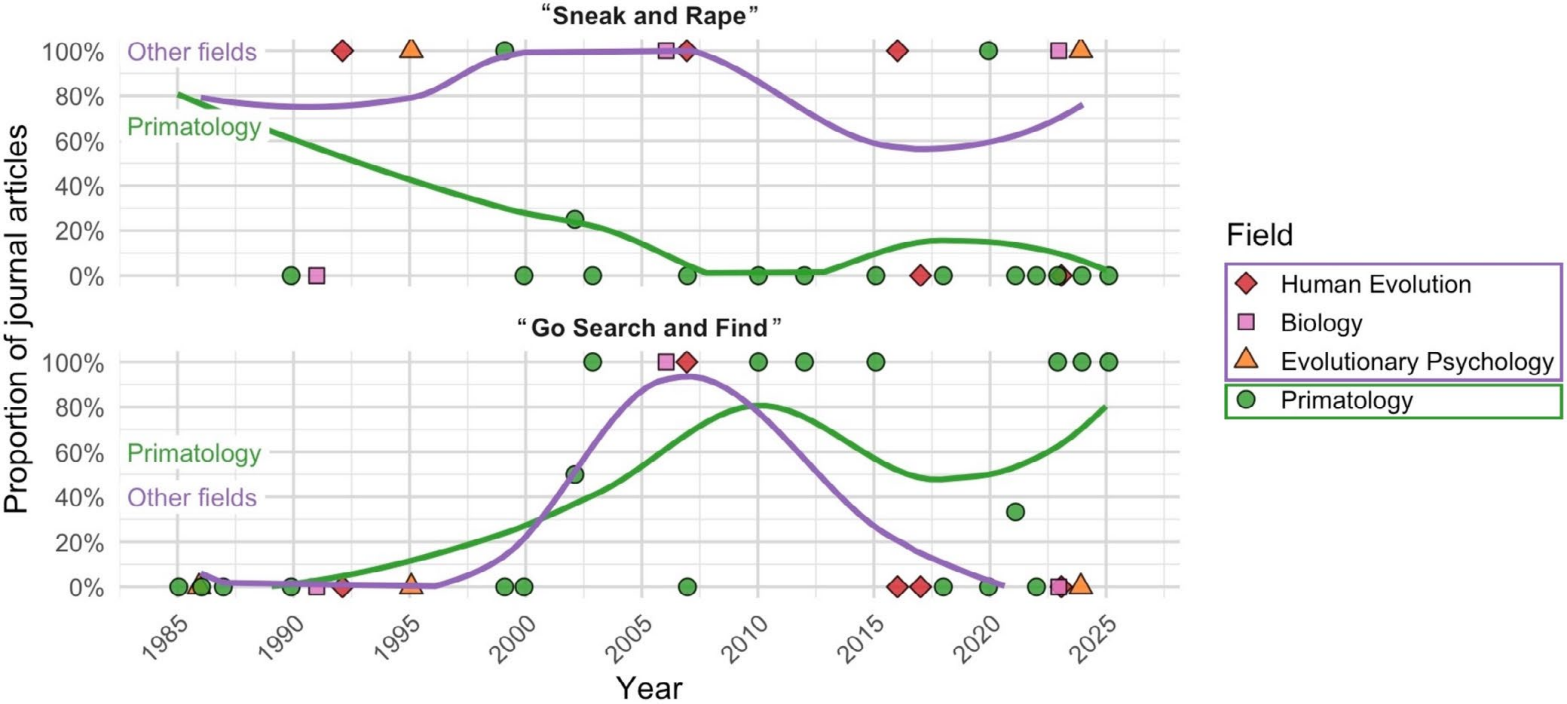
Percentage of peer‐review journal articles by year and field that describe subadult/unflanged/smaller/subordinate/etc male orangutans as using the “sneak and rape” strategy versus “go search and find.”

Figure 2 visualizes temporal shifts in terminology within the 39 peer‐reviewed journal articles that characterize orangutan alternative reproductive strategies. Notably, only five journal articles in primatology prior to 2002 describe this group of males as subadults who “sneak and rape” (SR) (Galdikas 1985a, 1985b; Maggioncalda et al. 1999; Schürmann and van Hooff 1986; Sugardjito et al. 1987). The remaining peer‐reviewed primatology articles before 2002 that discussed forced copulations adopted a nuanced approach describing 'subadult' males as a group that force copulates more often than 'adult' males, but also noted that these 'subadult' males may mate without force and that 'adult' males also may engage in forced copulations (FC1).

**FIGURE 3:**
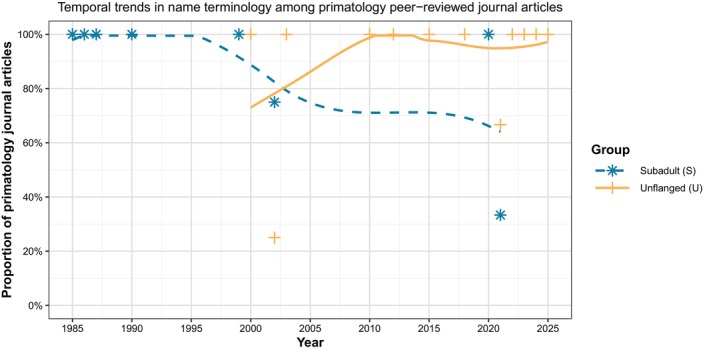
Percentage of all publications in peer‐ review primatology describing sexually active male orangutans without secondary sexual characteristics as either “subadult” or “unflanged.”

Around 2002, the literature shifted: the term “subadult” was replaced by “unflanged” (Figure 3) and only one peer‐reviewed article refers to these males as using a strategy of “sneak and rape,” and it is a study on snub‐nosed monkeys ( *Rhinopithecus bieti* ). Instead, the peer‐reviewed primatological literature categorized these males as unflanged and using either the strategy of “Go Search and Find” (GSF) or being described within the aforementioned nuanced FC1 framework.

**FIGURE 4:**
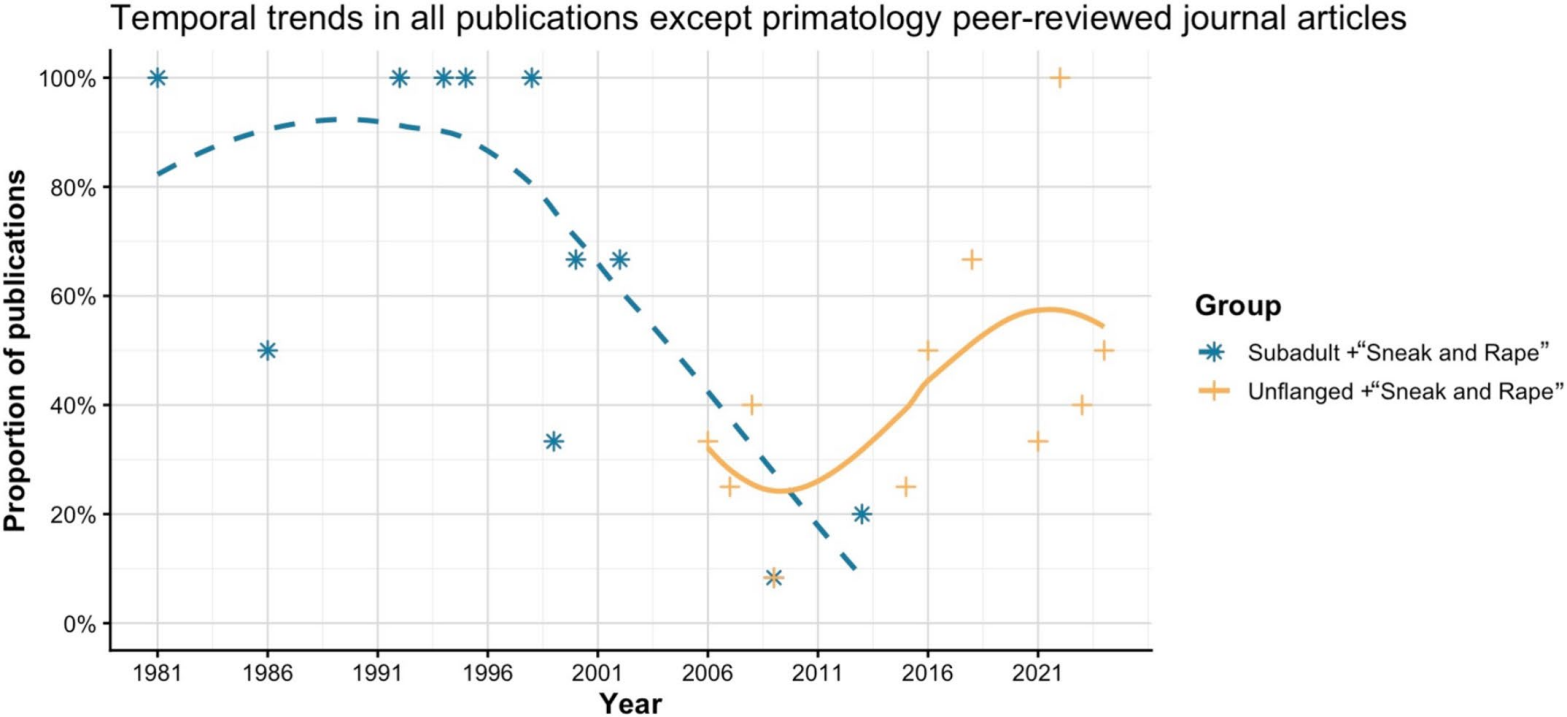
Percentage of all publications (excluding peer‐review primatology) describing sexually active male orangutans without secondary sexual characteristics as either “subadult” or unflanged and using the “sneak‐rape” terminology.

Figure 4 excludes the peer‐reviewed primatology literature to present an expanded dataset of all other academic publications, including non‐primatological peer‐reviewed publications and books, book chapters, dissertations, and theses that are both primatological and non‐primatological. The blue line in Figure 4 representing “subadult” “sneak and rape” remains visible over time and continues past 2002, indicating that this terminology continues to appear across the broader scholarly literature. This persistence is driven primarily by theses, book chapters, and publications in fields outside of primatology such as evolutionary psychology and human evolutionary biology. Interestingly, a yellow line in Figure 4 highlights publications describing males with updated name terminology, changed from 'subadult' to unflanged, but retaining the terminology of “sneak and rape” behavior.

*Result 2: “Subadult” and* “sneak and rape” *are referenced in publications that make arguments about the evolution of human behavior, in particular male aggression and female vulnerability*.

Results indicate that there are 21 instances in the literature in which descriptions of “subadult” or unflanged male orangutans employing a strategy of “sneak and rape” appear after the year 2002. All instances involve papers that are not original research on orangutan bimaturism and/or reproductive behavior. These papers were frequently found in evolutionary psychology and the evolution of human behavior. When “sneak and rape” is directly employed in an argument in the evolution of human behavior literature, it is most often used to support one of three main arguments: (1) that males are aggressive and females are more vulnerable to sexual aggression when solitary, whether human or non‐human primate, (2) that females are more attracted to dominant, large, high‐status males, whether human or non‐human, and (3) that sexual aggression/harassment is disproportionately performed by non‐preferred, subordinate, small, or low‐status males, human or non‐human. The first argument type, asserting that males are aggressive and females are more vulnerable to sexual aggression when solitary, is found prior to 2002. It is included here because it underlies the subsequent argument types. Argument types 2 and 3, which assert that females prefer dominant, high‐status human males and that sexual aggression is disproportionately performed by subordinate or low‐status human males, appear consistently across the literature after 2002.

First, publications outside of primatology used a description of unflanged or 'subadult' males employing a strategy of “sneaking or raping” to support arguments about human females being more vulnerable to human male violence. For example, the book *Demonic Males* calls upon this idea to argue that men inherited a propensity for violence and dominance from a shared evolutionary ancestor with other great apes and that this legacy helps explain patterns of male aggression and patriarchy in human societies. The authors write, “for orangutans, while we may not yet understand why small males benefit reproductively from raping, we can at least surmise that females are likely to be raped because, commonly moving through the forest alone, they are vulnerable” (Wrangham and Peterson 1996, 151–152). Other literature draws upon primate evidence to suggest that ancestral hominin females' ability to resist male aggression was compromised by reduced social support given orangutans are less social, referring to forced copulations as “the rule” in orangutans, writing “virtually all matings by sub‐adult males, and about half of the matings by adult males, occur after a prolonged and sometimes brutal struggle in which the much larger male forces the female to submit” (Smuts 1995, 11). A legal article extends this logic, using orangutans as an illustrative example to argue that sexual coercion emerges predictably under certain biological and social conditions across primates, including humans, and that law fails when treating such violence as exceptional rather than structural, stating that “the female orangutan, whose solitary existence leaves her vulnerable to violent sexual attacks by male orangutans” (Baker 1999, 817). This draws directly from orangutans, “In a variety of primate species, females who are not claimed as property by males are vulnerable to male aggression…. Among wild orangutans, most copulations by subadult males and nearly half of all copulations by adult males occur after the female's fierce resistance has been overcome through aggression” (Baker 1999, 811). Across these examples, there is an implication that aggression is an innately male trait shared across species lines, made visible in orangutans by their lower levels of sociality.

Second, publications outside of primatology mobilize orangutan alternative reproductive strategies as evolutionary support for an argument that human women are more attracted to human men who are large, strong, dominant, etc. For example, a manuscript from the journal *Evolution and Human Behavior* writes “In many species, females prefer traits that function in contests… For example, dominant male orangutans are larger and possess cheek flanges, and females show greater willingness to mate with these males near ovulation. These ideas help explain the origin of women's preferences for traits such as size, muscularity and deep voices that have clear utility in contests. The idea that such traits should be especially good indicators of male quality also helps explain why women tend to prefer them more for sexual relationships and during the fertile phase of the ovulatory cycle” (Puts 2010, 165). In *Women After All*, Konner argues that women are biologically and socially predisposed to be more cooperative, empathetic, and nurturing than men who are predisposed to aggression, referencing that this comes from evolutionary origins. In making this case, he argues that orangutan females prefer “older, larger, more experienced males, but as she ranges through the forest she may encounter younger, more aggressive ones” (Konner 2015, 106). He goes on to write, in an exaggerated loaded manner, that “these oversexed, frustrated, bullying subadults may not take no for an answer, and although they may be only half the size of mature males, they can gang up on a victim and overpower her” (Konner 2015, 106). Papers and books use the “subadult” orangutan “sneak and rape” narrative to argue that human females prefer traits like large male size and muscularity, that males lacking these traits are less desired, and that these dynamics manifest as increased aggression. These claims are often linked to evolutionary explanations, sometimes drawing on orangutan bimaturism as a comparative example.

Third, several evolutionary psychology publications extend this argument by equating orangutan alternative reproductive strategies with human male strategies, suggesting that certain types of human males are more likely to exhibit sexual aggression. For example, “Drawing from an evolutionary perspective, some theorists have proposed that male rape might be an adaptive, conditional mating strategy, such as the ones employed by male scorpionflies and orangutans. Specifically, Thornhill and Thornhill (1983) argued that men may have evolved to rape (or be more likely to rape) when they are unable to obtain a mate through intrasexual competition” (Li et al. 2012, 5). As for the type of men who adopt a conditional strategy supposedly like an unflanged orangutan, two have been proposed: those with psychopathy, and those with childhood stress. Two sources reference unflanged males to argue that psychopathy is a conditional reproductive strategy in human men (Brazil 2022; Huppin et al. 2019). One is the Handbook of Sexual Assault and Sexual Assault Prevention, which argued “from a comparative EP [evolutionary psychology] perspective, psychopathy can be considered a psychological phenotype most closely resembling the fixed morphological phenotype of the small male orangutans” (Huppin et al. 2019, 27). Second, this argument is made more broadly about lower‐ranking, stressed, less‐desired human men. A chapter titled “Rape” in the book *Attraction, Love, Sex: The Inside Story* directly equates unflanged males forcing copulations with human men who “lack resources” and “are socially disenfranchised” so use “coercive sexual strategies” in noting “something similar [to unflanged orangutan males] may be true for humans” (LeVay 2023). The author goes on to speculate whether a human equivalent to the “stressed” morph of unflanged males may be the case for one of the most infamous rapists in human history, “It's interesting in this context that Genghis Khan grew up in conditions of considerable deprivation after the early death of his father” (LeVay 2023). However, this conflicts with recent research showing that the remains of flanged males display more severe stress markers in their dentition than their unflanged counterparts, indicating that unflanged males actually had *less* severe early life stress events when compared to flanged males (Kralick and McGrath 2021). Additionally, the “sneak and rape” terminology had been found to lack empirical evidence long before 2023. Thus, these quotes indicate that beyond 2002 when the “subadult” term and the “sneak and rape” framing were found to lack empirical evidentiary support, there remain publications outside of primatology which continue to reference a non‐empirically supported narrative in order to support arguments regarding the evolution of human behavior, in particular, male aggression.

## 4 Discussion

*The persistence of the* “sneak and rape” *framework in the broader scholarly literature does not reflect empirical evidence; primatology distinguishes male morphs without defining them by* the *presence or absence of sexual aggression*.

This study demonstrates a clear shift in how male orangutan bimaturism has been conceptualized within primatology. Imprecise terminologies such as “subadult” and “sneak and rape” have largely been replaced, with the term “subadult” consistently replaced by “unflanged” in the primatological peer‐reviewed literature. Shifts in behavioral terminology are more mixed: prior to 2002, “sneak and rape” was the most common framing in the peer‐reviewed literature, whereas after 2002, “go search and find” became more prevalent. The replacement of “subadult” with “unflanged,” and the abandonment of the “sneak and rape” framework within primatology reflects more than a terminological update; it corresponds to a growing body of empirical work demonstrating that male orangutan reproductive behavior is flexible, context‐dependent, and not reducible to discrete categories based on presence or absence of male aggression. By the early 2000s, evidence clearly showed that forced copulations are neither exclusive to unflanged males nor constitutive of a distinct reproductive strategy, undermining the empirical basis of earlier taxonomies. Males without their full suite of secondary sex characteristics are documented at ages well into their twenties, thirties, or even beyond (Utami Atmoko and van Hooff 2004; Wich et al. 2004; Knott and Kahlenberg 2007), and are found to successfully sire offspring in the wild (Utami et al. 2002; Goossens et al. 2006; Tajima et al. 2018; Banes et al. 2015; Scott et al. 2024), do not exclusively force copulations, at times mate cooperatively, and are not the only males reported to force copulations (Knott et al. 2010; Kunz et al. 2023). Thus, there is a second framework that has consistently persisted over time, describing subadult or unflanged males as sometimes engaging in forced copulations at higher rates than flanged males, while acknowledging that some copulations are unforced and that flanged males also force copulate.

Primatology has moved away from defining male strategies by presence or absence of sexual aggression. Scholars have argued that forcing copulations is not an unflanged strategy but a strategy any male orangutan might use in response to female resistance (Knott and Kahlenberg 2007; Knott et al. 2010). Further, the dominant terminology of “sit, call, and wait” versus “go, search, and find” has nothing to do with forced copulations. Thus, the persistent idea found in the secondary literature that there is a smaller morph of younger male orangutans who employ a reproductive strategy of forcing copulations simply does not reflect current primatological consensus or empirical evidence.

*The persistence of the* “sneak and rape” *framework is not a product of disciplinary lag alone, as the same publications that retain “sneak and rape” are found to update the name for these males from subadult to unflanged and sometimes even update the framing of flanged males from* “consort and combat” *to* “calling and waiting”.

While taxonomies of “subadult” and “sneak and rape” are no longer the prevailing ones in primatology due to their lack of empirical support, the latter of the two has not disappeared from the broader academic ecosystem. It persists primarily in literature outside of primatology in fields that study the evolution of human behavior and outside of peer‐reviewed journal articles, in books and theses. The continued presence of “sneak and rape” in the broader literature is not simply a matter of disciplinary lag as other updates have occurred; the term unflanged has been taken up and subadult has been dropped across this literature. Fourteen publications were found that use the term unflanged alongside the term “sneak and rape” (Figure 4). Most surprisingly of all, five publications were found to have taken up part of the call‐and‐wait and go‐search‐find framework shift but only the part of flanged males, the “call and wait,” while failing to mention “go search and find” for unflanged males and instead still referring to them by the framework of “sneak and rape.” All five (coded CWSR) used the term unflanged, demonstrating a clear example of the selective updating. Taken together, these cases show that elements of newer frameworks have been incorporated unevenly, with some components adopted while others are omitted rather than integrated as a whole. Given the lack of empirical support for the “sneak and rape” framing, its continued appearance after the term ceased to dominate the primatological literature, and the longstanding critiques of both “sneak” and “rape” as scientific descriptors, its persistence begs explanation.

*The pattern of selective uptake was found to be connected to broader narratives in the evolution of human behavior* *which naturalize male aggression and female vulnerability*.

One consistent context in which this framing continues to appear is in work addressing the evolution of human behavior, where orangutan mating systems are mobilized to support broader comparative narratives. In particular, the quote analysis identified instances in which “sneak and rape” framings were mobilized in support of arguments regarding the evolution of human male behavior, aggression, and sexual violence. Particularly, orangutan bimaturism appeared as evidentiary support around three arguments about human behavior: that male aggression is innate, that females preferentially select dominant high‐status males, and that sexual aggression is more likely among subordinate or less‐preferred males.

While these arguments do not necessarily engage in the naturalistic fallacy in assuming that discussing evolutionary origins or biological underpinnings of male violence justifies that behavior (several were overtly anti‐misogynist in their intention), the arguments made in these publications are examples of biological determinism. These articles are naturalizing behaviors by implying that “human behavior originates in and is dictated by biological entities or processes” (Greene 2020), and draw on orangutan behavior as an example of biology. Even when framed as explanatory rather than justificatory, and even when mobilized in service of explicit critiques of violence, these arguments nonetheless position male aggression and female vulnerability as outcomes that flow predictably and naturally from evolved biological strategies. In doing so, they narrow the range of possible explanations for human behavior while downplaying the roles of social structure, historicity, and variability within both human and non‐human primates. The reference to a group of male orangutans “sneaking and raping” is used in this literature to stabilize particular narratives about sex, power, and aggression as naturalized features of human evolution rather than as patterns that require ongoing empirical scrutiny and contextual explanation.

Arguments which naturalize male violence and female vulnerability to male violence function to reinforce and uphold patriarchal values. In particular, the “sneak and rape” framework as comparative evidence carries interpretive weight to reinforce stereotypes and naturalize male aggression and female vulnerability. As Kamath and Packer write in *Feminism in the Wild*, “mainstream scientific narratives tend to align with the social, economic, and political narratives of those who hold power in our societies” (Kamath and Packer 2025, 10). It can be more challenging to dismantle patriarchal perspectives when they are naturalized and validated through scientific narratives (Kamath and Packer 2025). This is widely the case across biological sciences, but primate studies in particular have long been mined to reinforce stereotypes of male dominance given their evolutionary proximity to humans (Haraway 1989; Hrdy 1999). Sex essentialist and determinist science has been observed to biologize male violence by attributing aggression to an innate “biological maleness” (Fuentes 2021; Ferguson 2021). Dominance, competition, and aggression are privileged behaviors that are overrepresented in evolutionary biology models, while cooperation, diversity, and non‐dominance are marginalized or dismissed (Roughgarden 2013). Animal societies are often used to model human gender differences, reinforcing binary constructs of dominant males and passive females (Birke 2010). Just as feminist scholarship has shown that females are not always passive but can exhibit dominant behavior (Bosley 2010; Haraway 1989; Sperling 1991), queer feminism might also help us recover the roles of non‐dominant male strategies from the pejorative ways in which they have been described.

*Clarifying the frameworks in primatology to describe orangutan bimaturism is vital to advancing a more accurate understanding of the breadth of within‐male variation in orangutans and more responsible comparative inferences in fields that draw on primate models, including the evolution of human behavior*.

Capturing the full breadth and diversity of male strategies within our evolutionary frameworks and critically interrogating our assumptions opens the window for more nuanced and accurate ways of framing behavior and biology. As Kamath and Packer write, it is challenging to be certain that a prevailing story is accurate without research that critically interrogates that narrative (Kamath and Packer 2025, 5). Kamath and Packer outline an example of a type of male Coho salmon characterized as “sneakers” and demonstrate how challenging accuracy of the framework is to establish given that the body of research on Coho salmon typically already assumes that these males are sneakers. However, considering alternatives to the sneaker framework reveals a range of possible evolutionary explanations. For example, “scientists might accept that females are perfectly okay mating with multiple types of males of either type, with no explanations of coercion or sneaking required… To get comfortable with this multitude of possibilities is to let go of the idea that there is a stable, ‘true’ scientific story about any given animal” (Kamath and Packer 2025, 9).

The term “sneaker” is limiting. First, the “sneaker” characterization implies deception, obscuring possibilities of honest non‐deceptive interactions in the animal world, and underestimates the ability for conspecifics to observe and identify one another (Roughgarden 2013, 101). Second, it carries implications of a transgressor without access and of a behavior that is deceptive. Since unflanged males have been described as “stealing matings”, and the word “steal” implies deceit towards a rightful owner, this framing raises critical questions: Whose ownership is assumed? Are flanged males the rightful owners of matings? Who are these males deceiving, and by hiding what exactly? The notion that flanged males possess an inherent entitlement to matings that can be “stolen” fails to account for the agency of female orangutans. Further, the phrasing of “stolen” matings runs the risk of oversimplifying the complexity of social dynamics within the genus. Recognizing the breadth of within‐male variation has been transformative for primatology. Making explicit the way current primatological framings recognize the breadth of male variation is essential for responsibilizing the field, given the disciplines that draw upon its findings, such as human evolutionary studies.

Primatology has already undergone a profound reimagining of male roles in orangutans; these males are no longer characterized as sneakers in the peer‐reviewed primatology literature, and it is time to fully acknowledge and make explicit the substantive change that has already occurred in primatology. Today, the field embraces a model that recognizes the diversity of male strategies as evolutionarily valuable and examines male variation without defining difference by presence or absence of aggression. The framing of “calling and waiting” vs. “searching and finding” has nothing to do with forcing copulations and serves as a more neutral way of describing male strategies. Presence or absence of long call production serves as a reliable differentiator of flange status that avoids the limitations of strategy‐based framings, and is key to distinguishing the “call and wait” vs. “go search and find” framework. There are many paths forward toward fully embracing in terminology what primatology has long documented: a range of variation among males. Lower‐ranking flanged males, unflanged males who are sometimes larger or preferred by females, past‐prime males, and males in the process of developing flanges all fit within this spectrum. There exists terminology that recognizes a breadth of within‐male variation as central to understanding orangutan behavior. By embracing this shift, we can move towards a more accurate and nuanced primatology—one that recognizes the profound diversity of male strategies and their evolutionary significance. Recognizing and explicitly articulating the breadth of diversity within males is essential for producing accurate and nuanced interpretations of primate behavior, and for the studies of human evolution which draw upon it.

## 5 Conclusion

While significant shifts in terminology have taken place around male orangutan variation, imprecise categories persist, even if underrepresented. The transition from the framing of “subadults” who “sneak and rape” to unflanged males who “go, search, and find” is a critical step. Given the continued surfacing of terms lacking empirical support within literature on human evolution that makes biological determinist claims of male aggression and female vulnerability, terminological clarity necessitates explicitness. Defining males by forcing or not forcing copulations makes it hard to explicitly acknowledge the breadth of male variation. However, embracing existing frameworks that offer a more nuanced understanding of orangutan male behaviors not only advances a richer understanding of orangutan behavior but clarifies the primatological evidence base that is drawn upon for evolutionary models of human sexuality as being representative of substantial within‐male diversity and variation.

## Author Contributions

**Alexandra E. Kralick:** conceptualization, investigation, writing – original draft, methodology, validation, visualization, writing – review and editing, formal analysis, project administration, data curation.

## Conflicts of Interest

The author declares no conflicts of interest.

## Supporting information


**Appendix S1:** Supporting Information.

**Table S1:** Six Part Code definition.

**Table S2:** Sample sizes.

## Data Availability

All publications coded for data analysis are included in a supplemental works cited bibliography. The Excel data file with each publication's code, field, and quotations used in the analysis is available to researchers upon reasonable request. Access is mediated to minimize the risk of quotations being interpreted outside of their original context of the full publication.

## Endnotes

^1^
Only wild studies of orangutan populations observe adult unflanged males well into their twenties, thirties, or beyond in age (Hurlbut et al. 2026; Pradhan et al. 2012). In zoos there are adolescents who exhibit flange development later than other males but all are still within typical range of age of puberty (Graham and Nadler 1990; Kingsley 1982; Maple 1980), referred to as ‘arrested adolescents’ (Maggioncalda et al. 2002; Thompson et al. 2012).

^2^
In the 19th and early 20th centuries, Westerners traveled to present‐day Indonesia to capture orangutans for zoos and collect orangutan remains for museums, including Alfred Russel Wallace (van Wyhe and Kjærgaard 2015), co‐developer of the theory of evolution by natural selection (Smith 2005; Whittaker et al. 2013). Wallace observed an adult male lacking secondary sexual characteristics and, puzzled by its smaller size, hypothesized it represented a different species from larger males (Wallace 1856). Similarly, Abbott, who collected orangutans for the Smithsonian between 1903 and 1907, described “large” and “small” species of orangutans, a distinction Lyon (1908) later associated with the presence or absence of cheek pads.

^3^
The site of Tanjung Puting was provisioning the orangutans with food, and provisioning is now known to cause behavioral changes, including increased rates of aggression (Fedigan 2010).

^4^
The literature critically interrogating disciplinary language in primatology ranges broadly, beyond the term “rape”, to characterize mating system terms, i.e., “harem,” human‐ primate relationships, including “weed species,” “agricultural pests,” and “crop‐raiding,” and taxonomic terminology, such as New World or Old World primates (see Haraway 1989; Fedigan 2010; Hill 2017, 2018; Maréchal and McKinney 2020; Riley and Bezanson 2018; Norconk et al. 2020; as reviewed in Strier 2021).
