# Supplementary material for: No More Sneaking Around: The Changing Terminology of Orangutan Male Reproductive Strategies
Source: Am J Biol Anthropol. 2026 Jul 16;190(3):e70252. doi: 10.1002/ajpa.70252 (PMC13373747; doi:10.1002/ajpa.70252)
Supplement: Supplementary file 2 — Table S1: Six Part Code definition. [file AJPA-190-e70252-s003.docx]

**Supplemental Table 1. Six Part Code definition**

|  | **SR** | **SH** | **FC** | **FC1** | **GSF** | **O** |
| --- | --- | --- | --- | --- | --- | --- |
|  | **Sneak/Rape** | **Sexual Harassers** | **Force Copulations** | **Higher Rates of Forced Copulations** | **Go Search Find/No Long Calls** | **Other** |
| **Code Definition** | Refers to the evolutionary strategy/tactic of unflanged (or subadult, arrested, adolescent, smaller, undeveloped, subordinate, alterative, or other) morph of orangutans as… | | | | | |
|  | sneak and rape or rape or sneakers | sexual harassers who are coercive, bullies, or otherwise sexually aggressive | forcing copulations (may use euphemism like uncooperative or involuntary) to define these males | exhibiting higher rates force copulations, but qualifies that flanged males also force so it’s not only an unflanged behavior even if exhibited by them more and/or unflanged males do not always force | that of go-search-find or search-and-find or describes that (travels farther and/or does not long call) | an evolutionary strategy that is different from those others described, for example sociality or dietary ecology |
| **Inclusion Criteria** | Only mentions terms in code definition when describing the unflanged males or uses it to differentiate the morphs without qualification | | | forced copulation are what differentiates them but qualifies it as only mostly by the unflanged males, either that flanges males do it too or unflanged males don’t always | describes the difference between the morphs as about unflanged males traveling longer distances and/or flanged males being the ones who make long calls | any evolutionary strategy that differentiates the morphs and or may not relate to reproduction but cannot be about forcing copulations, travel distance, or long calls |
| **Exclusion criteria** | Not described as a strategy or tactic that differentiates the types of males | | | | | |
| **Typical exemplars** | “rape is a characteristically subadult male behavior”  “a “sneak/rape” reproductive strareg) prevails for subadult males” | "Sexual harassment is  the predominant  feature of the mating  strategy that subadult  males pursue” | “Orangutans offer the clearest examples of alternative tactics among primates, as the propensity of males to engage in forcible copulation is associated with clear morphological variation in facial ornamentation” | “Flanged” males are the older (and thus typically most dominant), preferred by females, and have prominent fleshy cheek ornamentations that more younger “unflanged” males lack. While both types engage in forced copulation, across sites, unflanged males are more likely to engage in forcible sex (see overview in Knott, 2009).” | “flanged” male orangutans exhibit a “sit, call, and wait”  strategy centered on sedentary residency in an area, accompanied by long calling, while  “unflanged” males adopt a more mobile “search and find” strategy” |  |
| **Atypical exemplars** | “subordinate males use an alter-native strategy in an attempt to obtain a copula-tion and are often referred to as "sneaky copulators," for they attempt to sneak copulation when their risks from more dominant males are low” | “aggressive ones. These oversexed, frustrated, bullying… gang up and overpower her” | “subadult  males tend to be involved in uncooperative copulations” “These forced copulations seem to be performed primarily by a subset of males.” | “unflanged (‘subadult’) who therefore tend to resort to forced matings” | “which  are thought to represent two well defined mating strategies, “call and wait”  by flanged males, and “sneak and rape” by unflanged male” |  |
| **Close but no** | “subadult male orangutans (Pongo pyugmaeus) reports of  “rape” (forced copulation) in primates are infrequent” | “so providing an opening for the unflanged male as a  quiet, quick, opportunistic “sexual predator.” | forced copulation as orangutan strategy and 90% of unflanged mating is forced but doesn’t say unflanged strategy  “hat travel with dominant adult  males experience reduced rates of forced  copulation by subadult males” | “Mitani (1985b) found both  flanged and unflanged males to force copulations with flanged males doing so mainly when  not locally dominant.” | “Flanged males use “long calls” to  communicate their presence to females and deter lower‐ranking  rival males while attracting higher‐ranking males” |  |
