# Supplementary material for: No More Sneaking Around: The Changing Terminology of Orangutan Male Reproductive Strategies
Source: Am J Biol Anthropol. 2026 Jul 16;190(3):e70252. doi: 10.1002/ajpa.70252 (PMC13373747; doi:10.1002/ajpa.70252)
Supplement: Supplementary file 3 — Table S2: Sample sizes. [file AJPA-190-e70252-s001.docx]

Sample sizes

| **Group** | **Count** | **Year_Range** | **JA** |
| --- | --- | --- | --- |
| **U_GSF** | 38 | 2002–2025 | 11 |
| **U_FC1** | 29 | 2000–2025 | 9 |
| **U_FC** | 27 | 2005–2024 | 6 |
| **S_SR** | 23 | 1981–2013 | 11 |
| **S_FC** | 14 | 1978–2009 | 3 |
| **S_FC1** | 14 | 1985–2018 | 5 |
| **U_SR** | 14 | 2006–2024 | 5 |
| **S_SH** | 7 | 1987–2021 | 3 |
| **U_Other** | 7 | 2003–2021 | 2 |
| **O_SR** | 6 | 1986–2019 | 2 |
| **U_SH** | 6 | 2006–2022 | 2 |
| **O_FC** | 4 | 2003–2021 | 1 |
| **U_CWSR** | 4 | 2007–2015 | 1 |
| **O_FC1** | 3 | 1988–2012 | 0 |
| **S_GSF** | 3 | 2002–2013 | 1 |
| **S_Other** | 3 | 1974–1995 | 2 |
| **S_CWSR** | 1 | 2013–2013 | 0 |
